# Metformin improves FOXP3 mRNA expression through suppression of interferon gamma levels in pristane-induced murine models of lupus

**DOI:** 10.12688/f1000research.23471.2

**Published:** 2021-06-11

**Authors:** Stevent Sumantri, Mochammad Hatta, Rosdiana Natzir, Haerani Rasyid, Iris Rengganis, Muhammad Nasrum Massi, Andi Asadul Islam, Gatot Lawrence, Ilhamjaya Patellongi, Andi Fachruddin Benyamin

**Affiliations:** 1 Department of Internal Medicine, Universitas Pelita Harapan, Tangerang, Banten, 15811, Indonesia; 2 Department of Microbiology, Universitas Hasanuddin, Makassar, Sulawesi Selatan, Indonesia; 3 Department of Biochemistry, Universitas Hasnuddin, Makassar, Sulawesi Selatan, Indonesia; 4 Department of Internal Medicine, Universitas Hasanuddin, Makassar, Sulawesi Selatan, Indonesia; 5 Department of Internal Medicine, Universitas Indonesia, Jakarta Pusat, Jakarta, Indonesia; 6 Department of Surgery, Universitas Hasanuddin, Makassar, Sulawesi Selatan, Indonesia; 7 Department of Pathological Anatomy, Universitas Hasanuddin, Makassar, Sulawesi Selatan, Indonesia; 8 Department of Physiology, Universitas Hasanuddin, Makassar, Sulawesi Selatan, Indonesia

**Keywords:** systemic lupus erythematosus, pristane induced lupus, oral metformin, intraperitoneal metformin, AMPK/mTOR pathway

## Abstract

**Background:**  A recent study has indicated the potential of metformin therapy for lupus in animal models, but there has been no study evaluating the effect on pristane-induced lupus. This study aims to evaluate the effect of intraperitoneal versus oral metformin on interferon (IFN)-γ levels and FOXP3 mRNA expression on pristane-induced female BALB/c mice.

**Methods:** In total, 31 female BALB/c mice, aged 6 weeks, were intraperitoneally induced with 0.5 ml of pristane (2,6,10,14-tetramethylpentadecane). After 120 days, the mice were grouped and treated with various treatments: normal saline 100 MCL, oral metformin 100mg/kg-BW, or intraperitoneal metformin 100mg/kg-BW. After 60 days of treatment, all treatment groups were sacrificed, and kidney specimens prepared and stained using hematoxylin and eosin.

**Results:** IFNγ levels of saline controls vs. oral metformin group was 309.39 vs. 292.83 pg/mL (mean difference 16.56 pg/mL; 95% CI 0.74-32.37; p=0.042), and saline control vs. intraperitoneal metformin group was 309.39 vs. 266.90 pg/mL (mean difference 42.49 pg/mL; 95% CI 29.24-55.73 pg/mL; p<0.001). FOXP3 mRNA expression changes in saline controls vs. oral metformin group was 6.90 vs. 7.79-fold change (mean difference -0.89-fold change; 95% CI -1.68-(-0.11); p=0.03)  and in saline controls vs. intraperitoneal metformin group was 6.90 vs. 9.02-fold change (mean difference -2.12-fold change; 95% CI -2.99-(-1.25); p=<0.001). Correlation analysis of FOXP3 mRNA expression and IFNγ level changes revealed a Pearson correlation of -0.785 (p=0.001) and R2 value of 0.616 (p=0.001).

**Conclusion:** Metformin is a potential new therapy to reduce the levels of IFNγ and increase FOXP3 mRNA expression in mice models of systemic lupus erythematosus.

## Introduction

Systemic lupus erythematosus (SLE) is a complex systemic disease, which is defined by multiple organ damage and dysfunction resulting from auto-antibody generation and inherited immune system dysregulation ^1^. The complicated pathophysiology and clinical manifestations result in difficulty in reaching an effective and comprehensive management of this condition. Lupus treatment currently relies on immunosuppressants and corticosteroids to suppress the immune system and reduce disease activity. This strategy is not ideal; there are several types of patients who do not respond well to immunosuppression and this therapy also produces side effects, such as recurrent infection, bone density loss, sarcopenia and psychological disturbances. This has led to infection and cardiovascular comorbidity becoming the major cause of mortality related to SLE, and not the disease itself ^2^.

Recent studies on experimental models has shown that the key to effective SLE management doesn’t rely on suppression of immune system, but how to manage and balance the activity of several key players, such as T regulator (Treg), T autoreactive (Th17), B autoreactive, and B regulator lymphocytes ^3^. Inflammatory cytokines and cellular components, such as tumour necrosis factor (TNF)-α, type 1 and 2 interferons, B-lymphocyte stimulator and interleukin-10 has also been known to contribute to the development of auto-antibodies and immune complexes that destroy tissues, especially in the kidneys ^4– 6^. Recently the activity of interferon (IFN)-γ and Th1 cells has been the focus of several experimental and clinical studies, especially its relationship to the development of lupus nephritis and its effect on downstream T-helper cells, such as Treg and Th17 ^7– 9^.

Several studies has also shown the influence of oxidative stress from environmental exposure to the imbalance of Treg and Th1 cells, with subsequent effects on the elevation of IFNγ levels and the development of SLE in exposed murine models ^10^. Exposure to reactive oxygen species is known to disrupt mitochondrial potential balance and activate the mTOR (mammalian Target of Rapamycin) metabolism regulation pathway by suppressing AMPK (Adenosine Monophosphate Kinase). This in turn results in a preference of Th1 pathway activation rather than Treg ^11– 13^.

Metformin, an old anti-diabetic drug with a reputable safety profile, recently has been known to be able to regulate the AMPK/mTOR pathway and from several studies has been shown to be able to regulate several autoimmune-, inflammation-, malignant- and aging-related conditions ^14, 15^. Studies on mice models of rheumatoid arthritis, autoimmune encephalitis and lupus nephritis has also shown this drug’s ability as a potential therapy of autoimmune disease ^11, 16^. This study aims to evaluate the effect of intraperitoneal versus oral metformin in decreasing IFNγ and increasing FOXP3 mRNA expression levels on pristane-induced female BALB/c mice, as there no studies that have evaluated the route of metformin delivery, especially on an environmentally induced model of lupus nephritis.

## Methods

### Animal models

In total, 30 female BALB/c mice, aged 6 weeks and weighing approximately 25 grams, were purchased from Universitas Hasanuddin Makassar (Indonesia) and then maintained at the Animal Unit of the Molecular Biology Laboratory of Universitas Hasanuddin Makassar from January 2018. The mice was kept in a temperature controlled housing according to their study group, food and water was provided freely. The number of mice for intervention study was determined using Federer formula for 5 groups. Efforts was made to minimize suffering, such as minimal handling, less frequency of venous sampling, adequate space for living and no other experimentation or pain inducing procedures.

After 2 weeks of acclimatization the mice were then randomly divided into five groups (6 mice/group): group 1, normal control; group 2, SLE model; group 3, normal saline; group 4, oral metformin; and group 5, intraperitoneal metformin groups. Four of the groups (all apart from normal control) were induced with 0.5 ml of pristane (2,6,10,14-tetramethylpentadecane; Sigma Aldrich) intraperitoneally. The normal control group was injected with normal saline 0.5 ml intraperitoneally as a control group. After 120 days, groups 1 and 2 were sacrificed using chloroform euthanasia methods (dose of 20 g/m3 in a closed chamber system). Kidney specimen was then fixed with neutral buffered formalin (NBF) 10%, prepared in paraffin block, sliced to 2μM thickness and then stained with haematoxylin and eosin (H&E).

All intervention, analysis and reporting conducted in this study follows the ARRIVE guidelines for animal studies. Ethical approval for animal studies was obtained from Universitas Hasanuddin’s Health Studies Ethical Committee, with protocol number UH17030037. Care and intervention conducted in the research animal, refers to Indonesian National Guidelines on Health Research Ethics and Indonesian Food and Medicine Regulatory Body Guidelines on Good Clinical Practice ^17^.

### Intervention

After 120 days, the intervention groups (groups 3–5) were given therapy every morning in the animal laboratory according to their designation: group 3, given 100 mcl normal saline via oral gavage once daily; group 4, given 100 mg/kg body weight of metformin diluted in 100 mcl normal saline via oral gavage once daily; group 5, given 100 mg/kg body weight of metformin diluted in normal saline via intraperitoneal injection once daily. Treatment lasted for 60 days and at the end of the period all three groups were sacrificed, kidney specimen fixated with NBF 10%, prepared in paraffin block and then stained with H&E.

### Cytokine and mRNA expression measurement

Samples for IFNγ and FOXP3 mRNA measurements was collected from tail vein sampling (0.1–0.2 ml for each sample). IFNγ was measured using the murine IFNγ ELISA kit from Abcam (ab100689) and read using ELISA Reader 270 with 450 nm wavelength (Biomerieux, France).

Total RNA was isolated from using the Qiagen RNeasy Micro Kit (DNA Genotek, Qiagen) according to the manufacturer’s instructions. Complementary DNA synthesis was performed by using iScript™ Advanced cDNA Synthesis Kit for RT-qPCR (Bio-Rad). Using cDNA synthesized from 150 ng of total RNA as a template for one amplification, real-time reverse transcriptase (RT)-PCR (CFX Connect system; Bio-Rad) was performed using SYBR® Green PCR Master Mix one step according to the instructions provided (Bio-Rad). Final reaction volume was 20 μL, and included 2 μL cDNA, 10 μL SYBR Green Master Mix, 0.5 μL each of the forward and reverse primers (10 pmol), and 7 μL nuclease-free water. Amplification conditions used for qPCR were: 95°C for 2 minutes, followed by 40 cycles of denaturation and annealing/extension cycles at 95°C for 5 seconds and 60°C for 30 seconds.

The glyceraldehyde-3-phosphate dehydrogenase (GAPDH) gene was used as an internal control for normalization, GAPDH primer, forward (5’GAAGGTGAAGGTCGGAGT-3’) and reverse (5’-GAAGATGGTGATGGGATTTC-3’). Fold change was determined by the ΔΔCt method. All measurements were conducted as per manufacturer’s instructions and repeated three times to ensure the validity. Target protein concentration was read as picogram/millilitre and mRNA expression as fold change.

### Histopathological analysis

Kidney specimens were examined by two independent and blinded histopathologists experienced in evaluating murine renal samples. Glomerular scores were evaluated by measuring the level of destruction on 50 glomerular units in each mouse and scored as 0 = normal, 1 = mesangial expansion, 2 = endocapillary proliferation, 3 = capillaritis or necrotic changes and 4 = crescents. Interstitial scoring was measured by evaluating 50 high power fields and scored as 0 = no interstitial involvement, 1 = <25% interstitial involvement, 2 = 25–50% involvement and 3 = >50% involvement ^18^.

### Statistical analysis

Statistical analysis (SPSS Statistics ver. 20, IBM) was done by measuring mean difference (one way ANOVA with Tukey’s post test for multiple comparisons) to evaluate the difference in IFNγ levels, FOXP3 mRNA fold change and histopathological scores between the three intervention groups (comparing saline control, oral and intraperitoneal metformin groups). Mean difference analysis using t-test was done for the proof of concept group (comparing normal control and SLE model groups). Correlation analysis was also done to evaluate the relationship between IFNγ and FOXP3 mRNA changes to determine the strength of the causality. All tests were reported with 95% confidence interval, standard error and statistical significance score (p<0.05).

## Results

Two female mice expired in the adaptation period; therefore, only 29 mice entered the intervention period and finished the experiments without problems, analysis was done with 5 mice from each group (n=25).

**Figure 1.**
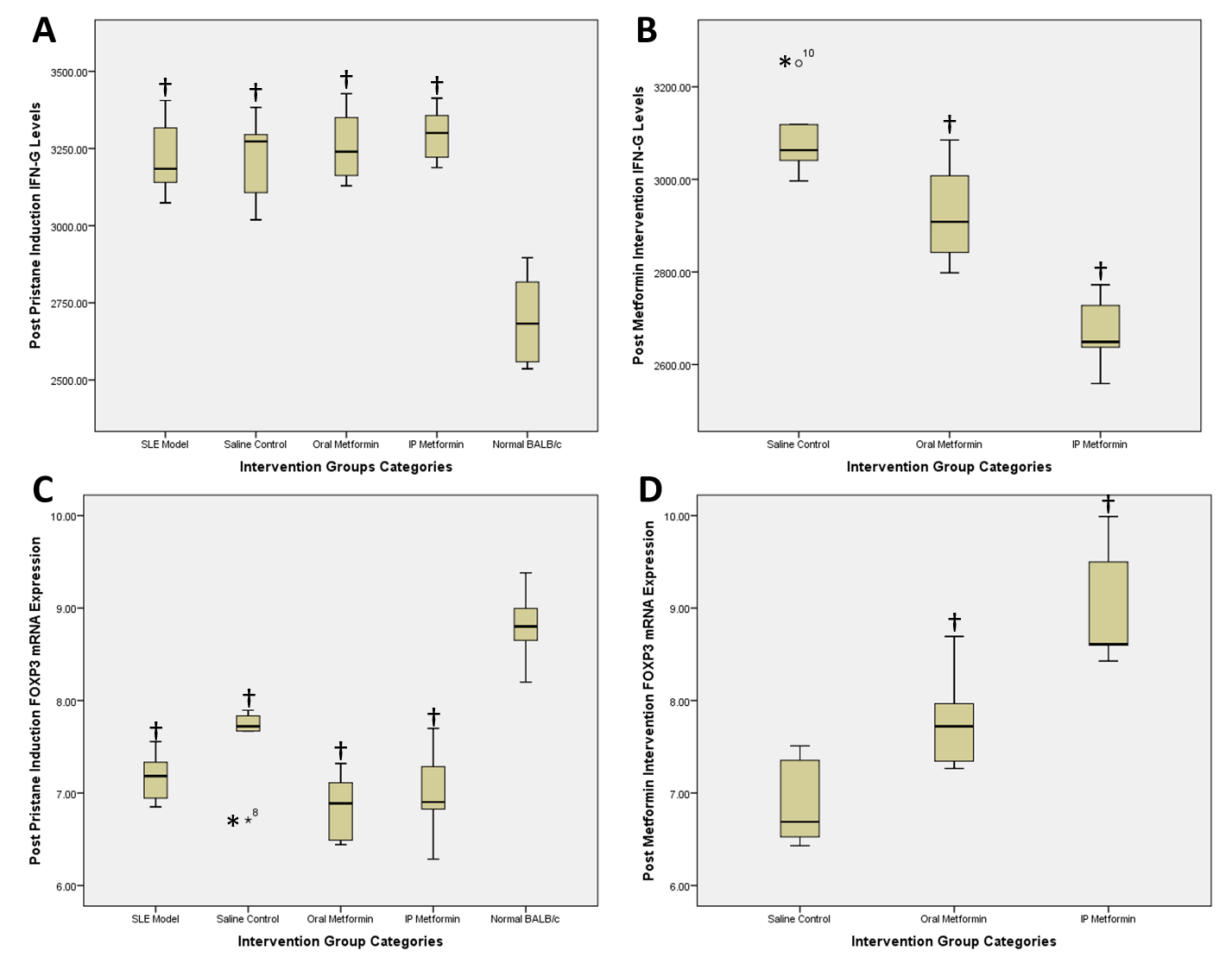
Post pristane induction and post metformin intervention IFNγ levels ( **A** and **B**) and FOXP3 mRNA expression ( **C** and **D**). †statistically significant compared to control groups; *outliers.

Groups 1 and 2 (normal control and SLE model) were sacrificed at the end of the initial 120 day induction period and IFNγ and FOXP3 mRNA expression changes are detailed in Table 1. The starting level of IFNγ shows no difference between normal control and SLE model groups (269.60 vs. 281.12 pg/mL; mean difference 11.52 pg/mL; 95% CI -17.47 – 40.52 pg/mL; p=0.386). Post-induction with intraperitoneal pristane (for group 2 only), there was a difference in IFNγ levels between normal control and SLE model groups (269.82 vs. 322.42 pg/mL; mean difference 52.59 pg/mL; 95% CI 31.23-73.96 pg/mL; p<0.001) ( Figure 1A). The expression of FOXP3 mRNA at baseline shows no difference between normal control and SLE model groups (8.87 vs. 8.86-fold change; mean difference -0.00-fold change; 95% CI -0.78-0.77; p=0.983), while post-pristane induction there was a mean difference of -1.63-fold change of mRNA FOXP3 expression between groups (8.80 vs. 7.17-fold change; 95% CI -2.17 – 1.09-fold change; p<0.001) ( Figure 1C).

**Table 1.** Comparison of IFNγ levels and FOXP3 mRNA expression before and after induction with intraperitoneal pristane. Data are presented as mean (95% confidence interval). SLE, systemic lupus erythematosus; PI, post induction.

	IFNγ (pg/ml)		Mean difference	p	FOXP3 mRNA (fold change)		Mean difference	p
	Baseline	PI			Baseline	PI		
**Normal BALB/c** **(n=5)**	269.60 (254.08-285.12)	269.82 (250.27-289.37)	-0.24 (-24.13-23.68)	0.98	8.87 (8.43-9.31)	8.80 (8.26-9.34)	0.07 (-0.51-0.65)	0.753
**SLE model** **(n=6)**	281.12 (249.85-312.39)	322.42 (305.70-339.15)	-42.39 (-83.26-6.66)	0.052	8.86 (8.04-9.69)	7.17 (6.81-7.53)	1.69 (0.63-2.75)	0.011

Groups 3–5 (normal saline, oral metformin and intraperitoneal metformin) entered the 60 days of intervention period. IFNγ and FOXP3 mRNA expression changes can be seen in Table 2. A one-way between subjects ANOVA was conducted to compare the effect of the aforementioned intervention. There was a significant difference found between post-intervention groups regarding the level of IFNγ (F (2,12) = 22.611; p<0.001) and FOXP3 mRNA expression (F (2,12) = 16.330; p<0.001). Post-hoc analysis using Tukey HSD resulted the following results for mean difference between groups. Comparison between saline control and oral metformin groups resulted in IFNγ levels of 309.39 vs. 292.83 pg/mL (mean difference 16.56 pg/mL; 95% CI 0.74-32.37; p=0.042; Figure 1B). Comparison between saline control and intraperitoneal metformin groups resulted in IFNγ levels of 309.39 vs. 266.90 pg/mL (mean difference 42.49 pg/mL; 95% CI 29.24-55.73 pg/mL; p<0.001; Figure 1B). Comparison between oral and intraperitoneal metformin groups resulted in IFNγ level changes of -33.34 vs. -62.70 pg/mL (mean difference 29.35 pg/mL; 95% CI -52.08 – (-6.63); p=0.004; Figure 1B).

**Table 2.** Comparison of IFNγ levels and FOXP3 mRNA expression before and after intervention with metformin in pristane-induced BALB/C mice. Data are presented as mean (95% confidence interval).

	IFNγ (pg/ml)		Mean difference	p	FOXP3 mRNA (fold change)		Mean difference	p
	Before	After			Before	After		
**Saline control** **(n=6)**	321.54 (303.11-339.97)	309.39 (297.23-321.55)	12.14 (-17.3-41.61)	0.316	7.56 (6.96-8.17)	6.90 (0.09-1.22)	0.66 (0.09-1.22)	0.031
**Oral metformin** **(n=6)**	326.18 (310.57-341.78)	292.83 (278.18-307.48)	33.34 (10.74-55.94)	0.015	6.85 (6.37-7.32)	7.79 (7.08-8.51)	-0.94 (-1.53-(-0.35))	0.011
**IP metformin** **(n=6)**	329.61 (318.07-341.14)	266.90 (256.58-277.22)	62.70 (47.28-78.12)	<0.001	6.99 (6.34-7.65)	9.02 (8.17-(-1.09))	-2.02 (-2.95-(-1.09))	0.04

FOXP3 mRNA expression changes between saline control compared with oral metformin revealed 6.90 vs. 7.79-fold change (mean difference -0.89-fold change; 95% CI -1.68-(-0.11); p=0.03; Figure 1D), while between saline control and intraperitoneal metformin there was a 6.90 vs. 9.02-fold change (mean difference -2.12-fold change; 95% CI -2.99-(-1.25); p=<0.001; Figure 1D). Comparison of FOXP3 mRNA expression between oral and intraperitoneal metformin groups was -0.94 vs. (-2.02) fold change (mean difference 1.07-fold change; 95% IC 0.16-1.99; p=0.027; Figure 1D).

**Figure 2.**
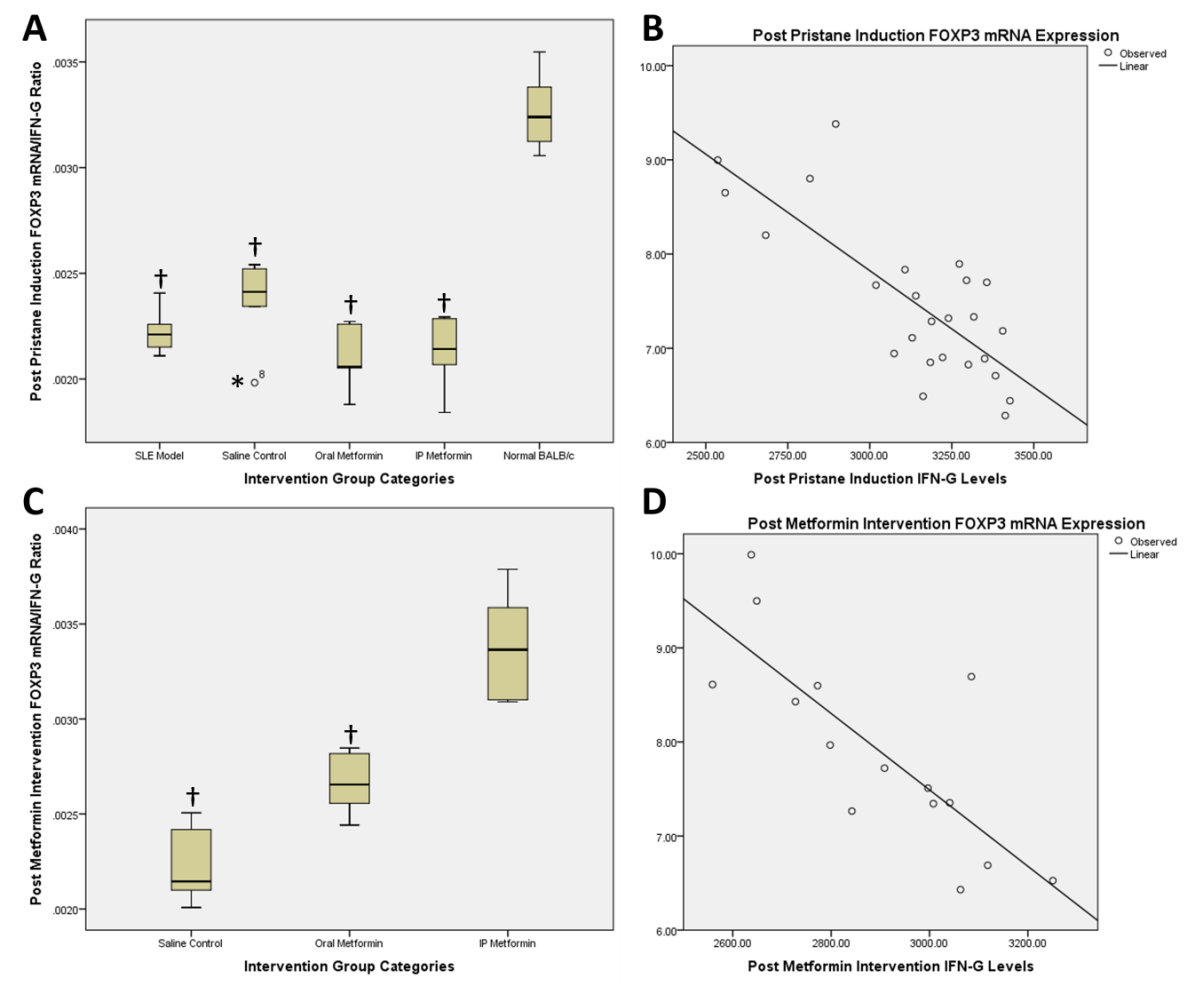
Post pristane induction FOXP3 mRNA/IFN-gamma ratio and correlation scatter plot ( **A** and **B**) and post metformin therapy FOXP3 mRNA/IFN-gamma ratio and correlation scatter plot ( **C** and **D**). †statistically significant compared to control groups; *outliers.

Ratio of FOXP3 mRNA expression and IFNγ levels represents the balance between Treg (anti-inflammatory) and Th1 (pro-inflammatory) activity. Post pristane induction in BALB/c mice showed a ratio of 0.002 vs. 0.003 (mean difference -0.001; 95% CI -0.001 – (-0.0008); p<0.001) in SLE model compared to normal BALB/c group ( Figure 2A). Correlation analysis of FOXP3 mRNA expression and IFNγ level changes pre and post induction with pristane in the five groups revealed a Pearson correlation of -0.776 (p<0.001) and R ^2^ value of 0.602 (p<0.001) ( Figure 2B).

Post intervention, comparison between saline control with oral metformin groups was 0.0022 vs. 0.0027 (mean difference -0.0004; 95% CI -0.0007 – (-0.0001); p=0.008; Figure 2C), while between saline control and intraperitoneal metformin groups was 0.0022 vs. 0.0034 (mean difference -0.001; 95% CI -0.0015 – (-0.0007); p<0.001; Figure 2C). Comparison between oral and intraperitoneal metformin groups revealed 0.0027 vs. 0.0034 (mean difference -0.0007; 95% CI -0.0011 – (-0.0003); p=0.002; Figure 2C). Correlation analysis of FOXP3 mRNA expression and IFNγ level changes between post induction and post treatment with metformin revealed a Pearson correlation of -0.785 (p=0.001) and R ^2^ value of 0.616 (p=0.001) ( Figure 2D).

**Figure 3.**
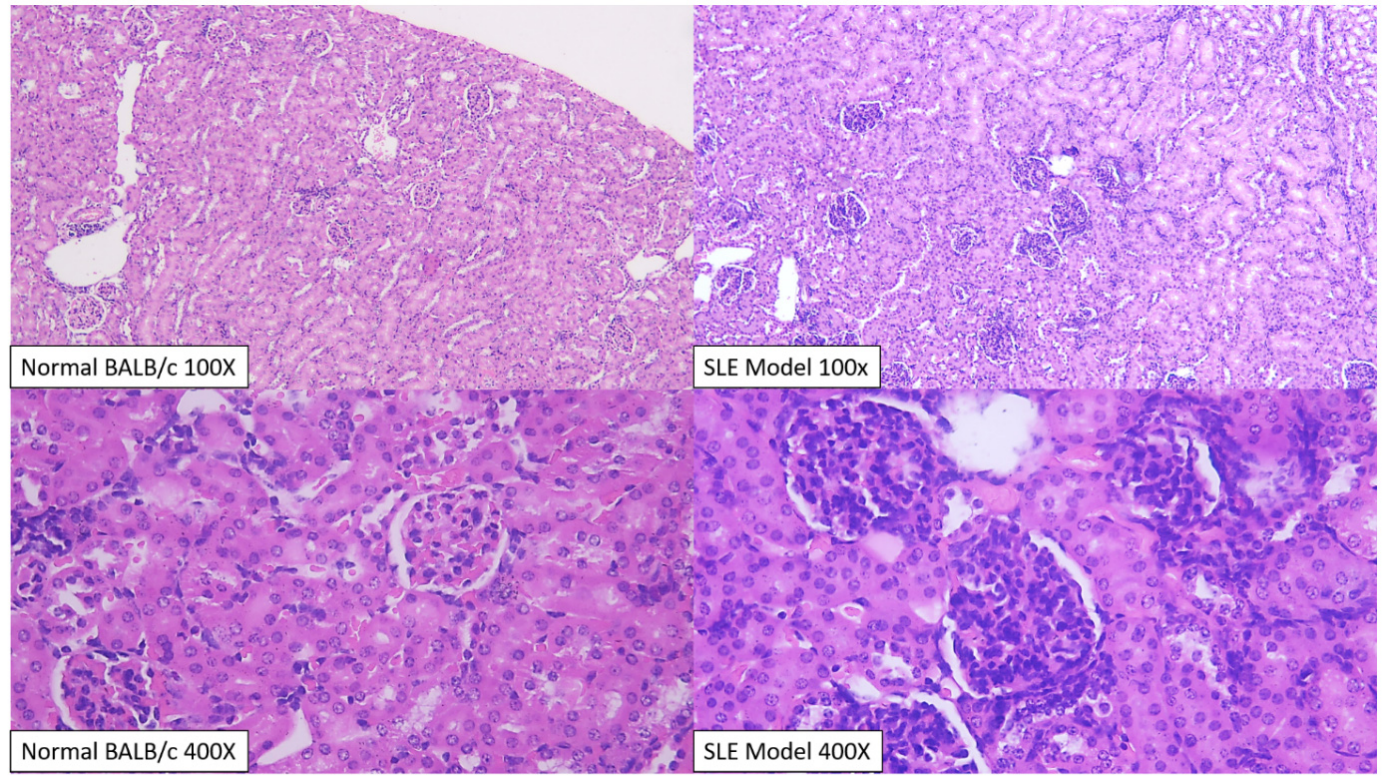
Kidney of BALB/c mice stained with hematoxylin and eosin, before intervention. Analysis on normal BALB/c control kidney (upper and lower left) revealed mild tubulo-nephritis changes, with minimal mesangial expansion, endocapillary proliferation and capillaritis. Significant interstitial infiltration (25–50% field) only happens in one member of normal BALB/c group. In the SLE model group (upper and lower right), after induction with pristane, there was a significant change in the glomeruli, with dominant endocapillary proliferation and minimal mesangial expansion and capillaritis.

Histopathological analysis on kidney specimens resulted in a variable change in each intervention group ( Figure 3). Glomerular scoring comparison between BALB/c normal and SLE model groups revealed a score of 2.2 vs. 3.0 (mean difference 0.80; 95% CI 0.33-1.26; p=0.04). Interstitial scoring comparison between BALB/c normal and SLE model groups revealed a score of 1.20 vs. 1.40 (mean difference 0.20; 95% CI 0.83-1.23; p=0.667). Total histopathological scoring between the two groups revealed a score of 4.40 vs. 3.40 (mean difference 1.00; 95% CI -0.08 – 2.08; p=0.066).

**Figure 4.**
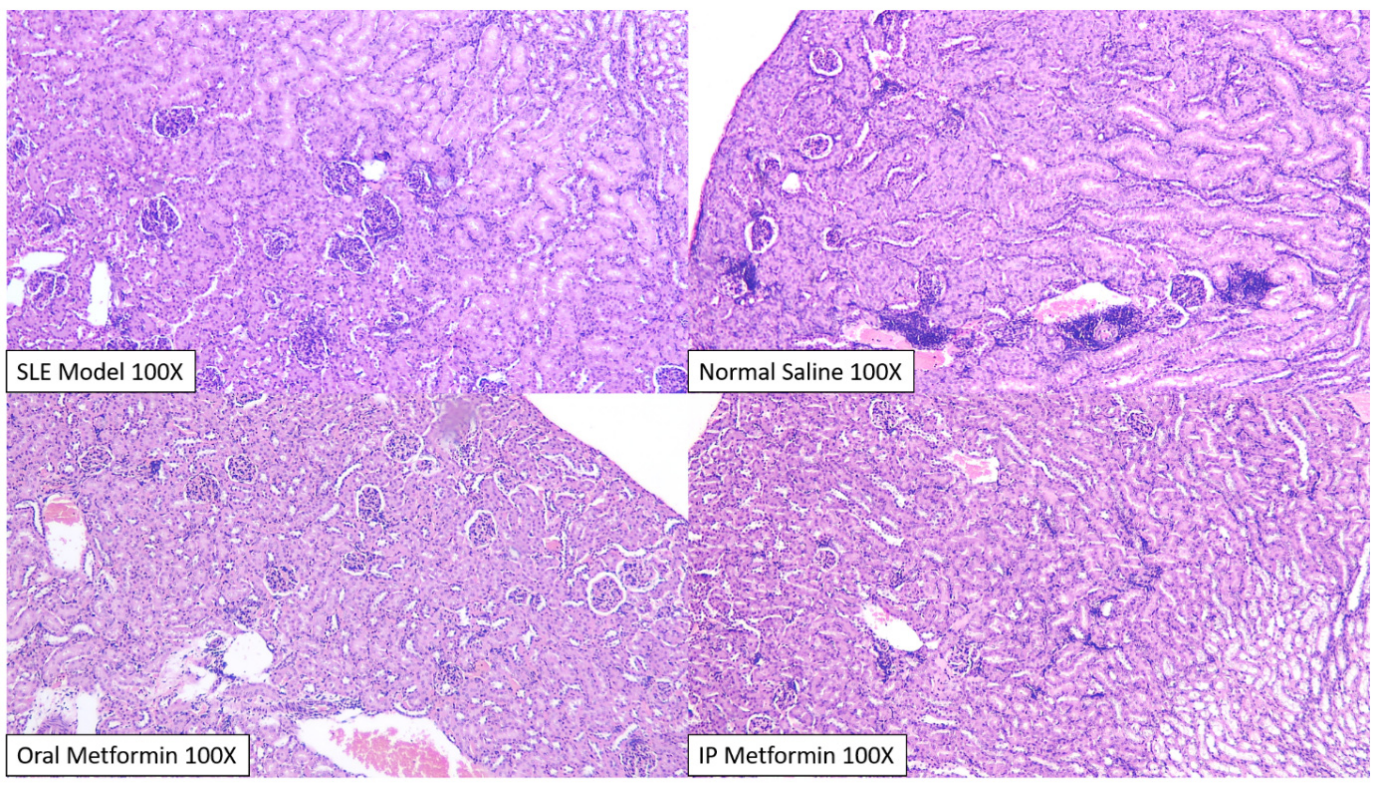
Kidney of BALB/c mice stained with hematoxylin and eosin, after intervention. In general, there was a widespread and significant glomerular and interstitial change across all groups, consistent with previous SLE models. Tubulo-nephritic changes significantly happens more than glomerulo-nephritis, although significant interstitial infiltration only happens in the normal saline group (upper right), especially in two members of its group. Qualitatively, two blinded pathologists concluded that the most severe changes happen in the normal saline group and the least severe in the intraperitoneal metformin group.

Histopathological scoring between intervention groups (normal saline, oral and intraperitoneal metformin) did not reveal significant differences, although qualitative analysis by blinded pathologists revealed difference in the degree of nephritis occurring in each group ( Figure 4).

## Discussion

SLE is a multifactorial inflammatory autoimmune disease, with clinical manifestations that involves various tissues and organs. The aetiology of this autoimmune condition is linked to dysfunctional B and T lymphocyte responses to environmental stimulus in a genetically susceptible individual, which in turn determines the immune response to various autoantigens and can cause tissue damage. The application of pristane, an aromatic hydrocarbon, has an advantage to genetically modified mice, because the ability of this model to mimic SLE in humans, which in a genetically susceptible individual is usually caused by environmental exposure. Pristane mouse models also enables researchers to evaluate pathophysiological changes in a timely manner, and to give a picture of the cellular mechanism involved in the development and progressivity of SLE ^12^.

In this study, we showed that after induction with pristane, there was a significant difference in the level of IFNγ in the normal BALB/c group compared to the SLE model (269.82 vs. 322.42 pg/mL; mean difference 52.59; 95% CI 31.23 - 73.96; p<0.001). Richards *et al.* ^12^ showed that IFNγ is an important component in the development of lupus nephritis in pristane-induced murine models. Induction with pristane in an IFNγ deficient mouse (IFNγ-/-) would not result in a change of renal pathology corresponding to lupus nephritis. Furthermore, in this model of IFNγ deficient mice, post pristane induction, no antibodies characteristically associated with lupus, such as IgG anti-ssDNA and anti-chromatin antibody, were found ^12, 19^. Chiche *et al.* ^19^ also noted that the activity of pathways related to IFNγ play an important role in the development of anti-dsDNA antibodies and the reduction of lymphocyte counts in patients with SLE.

Regarding the expression of FOXP3 mRNA, we showed that there was a significant difference between normal BALB/c compared to the SLE model group (8.80 vs. 7.17-fold change; mean difference -1.63; 95% CI 2.17 – 1.09; p<0.001). The expression of FOXP3 mRNA in pristane induced murine models is a marker for Treg cell activity. In a study by Peixoto *et al*., it was shown that on day 90 and 120 post induction there was a decrease in CD4+CD25+FOXP3+ (Treg) cell count in peripheral blood samples ^20^. These authors also showed that the reduction in Treg count was correlated with an increase in IFNγ (p=0.017), TNFα (p=0.043) and TGF-β1 (p=0.038). Furthermore, Kluger *et al.* also pointed out that the disturbances in Treg (FOXP3+) function contributes to the development of acute glomerulonephritis in pristane induced lupus ^21^.

In this study, metformin intervention, whether in oral form (309.39 vs. 292.83 pg/ml; mean difference 16.56; 95% CI 0.74-32.37; p=0.042) or intraperitoneal injection (309.39 vs. 266.90 pg/ml; mean difference 42.49 pg/ml; 95% CI 29.24-55.73; p<0.001) gave a significantly superior suppression of IFNγ levels. A previous study by Mardani *et al*. showed that an intervention with probiotics could reduce IFNγ and IL-17 levels in pristane induced murine lupus, followed by a reduction in autoantibodies such as ANA, anti-dsDNA and anti-RNP ^9^. Reduction of IFNγ levels could improve the outcome of lupus nephritis by inactivating B7/CD28 signalling pathway, which results in a reduction in ANA autoantibodies, IL-4 and IFNγ levels. The inactivation of B7/CD28 pathways also caused anergy, tolerance and apoptosis of T-cells, which results in a decrease of urine protein and immune complex deposition in the kidneys of pristane induced C57BL/6J mice ^22^.

This study revealed that the administration of oral and intraperitoneal metformin gave a significantly better suppression of IFNγ than placebo, in accordance to the study conducted by Yin *et al.* ^23^. In that study, intervention with metformin and 2-DG (2-deoxy-glucose) in B6.Sle1Sle2.Sle3 mice resulted in a suppression of IL-17 and IFNγ levels through a blockade on the glucose oxidation pathway. This blockade on the glucose oxidation pathway also normalized T-cell metabolism, which in turn suppresses the activation of CD4+ T-cells and returns the balance of pro/anti-inflammatory cytokines in mice models ^10^.

Regarding FOXP3 mRNA expression, this study showed that intervention with metformin via oral (6.90 vs. 7.79-fold change; mean difference -0.89; 95% CI -1.68 – (-0.11); p=0.03) or intraperitoneal route (6.90 vs. 9.02-fold change; 95% CI -2.99 – (-1.25); p<0.001) gave a significantly superior increase in FOXP3 mRNA expression compared to saline control. We also showed that there was a strong significant inverse correlation between the increase of FOXP3 mRNA expression and decrease of IFNγ resulting from metformin intervention (R=-0.785; p=0.001). Furthermore, it seems that the reduction of IFNγ explains the increase in FOXP3 mRNA expression rather strongly, as shown by the R ^2^ value of 0.616 (p=0.001). The above result was consistent with several studies in pristane induced models; a decrease in FOXP3+ T-cells and increase in CD4+CD69+ T-cells coincide with an increase in IFNγ levels in intraperitoneal fluid ^20, 23^.

To the best of our knowledge, this is the first study that evaluates the effect of metformin on the expression of FOXP3 mRNA in lupus, although it is also known that metformin has the ability to induce AMPK pathway activity and suppress mTOR signalling ^24, 25^. Metformin has also been known to be able to improve disease activity index, histological and inflammatory profiles in several other autoimmune models, such as inflammatory bowel disease ^26^ and autoimmune insulitis ^14^ through the modulation of AMPK-mTOR pathway and the resulting changes in IL-17, IFNγ, IL-10 and FOXP3 associated cytokines and cells.

Intraperitoneal route of metformin gave a superior effect on the suppression of IFNγ levels and increasing FOXP3 mRNA expression compared to the oral route, and to the best of our knowledge this was the first study that observed this effect in pristane induced murine model of lupus. A study by Dowling *et al*. on NOD/SCID mice revealed that plasma levels of metformin were higher via intraperitoneal than oral route (145 uM vs. 77 uM; range 65.8-214.7 uM vs. 41.6-99.0 uM) ^27^. Thus it is concluded that intraperitoneal metformin gave a higher suppression of IFNγ and increase of FOXP3 mRNA expression through an plasma level rather than an oral route. In addition, a study by Wang *et al*. in a scleroderma model has also shown the ability of intraperitoneal metformin to dose dependently reduce IL-17A levels and RORγt expression and increase FOXP3 mRNA expression ^28^.

Although this study was able to prove that there was a characteristic change in accordance to lupus nephritis in pristane induced models compared to normal BALB/c, subsequent therapy with metformin failed to produce a statistically significant score change. However, qualitative analysis by blinded pathologists has confirmed that there was at least a difference in renal changes that showed better results in intraperitoneally treated mice compared with oral metformin and placebo control. This result could be caused by a short period of intervention; a longer treatment time could possibly result in a significant difference in renal scoring.

### Limitations

We did not perform an evaluation of autoantibodies related to SLE, such as anti-dsDNA, anti-Sm and anti-RNP1. However, several murine studies has confirmed the ability of pristane induced BALB/c in producing related auto-antibodies ^29, 30^. Our research also did not evaluate the antibody response to metformin therapy; however several studies has shown the ability of metformin in reducing autoantibodies related to SLE ^10, 22^. We also did not evaluate the expression of mRNAs related to IFNγ, but several studies has shown that Th1 activity is closely related to IFNγ levels ^8, 31, 32^. Furthermore it has been recently suggested that the cytokine balance could play an important role in determining active T-cell subsets, changing the phenotype of peripheral T-cells and contributes to the pathogenesis of lupus ^13, 33^.

## Conclusions

A murine model of SLE by pristane induced female BALB/c mice could be used to represent a model of lupus similar to the human condition. The increased activity of Th1 and reduced activity of Treg, in this study represented by pro-inflammatory IFNγ levels and FOXP3 mRNA expression, has proven to be related to the development of lupus nephritis. Metformin is a potential new therapy to reduce the levels of IFNγ and increase FOXP3 mRNA expression in SLE and in turn inhibits the development of glomerulonephritis. Intraperitoneal metformin, intravenous in humans, could provide a novel route of administration to improve the effect of metformin on lupus patients.

## Data availability

### Underlying data

Open Science Framework: Metformin on Pristane Induced Lupus, https://doi.org/10.17605/OSF.IO/S9GRP ^34^.

This project contains the following underlying data:

- IFNγ levels for all mice pre and post intervention;
- FOXP3 expression fold change for all mice pre and post intervention;
- Interstitial and glomerular scoring for all mice; and
- Uncropped, unedited kidney images for all mice.

Data are available under the terms of the Creative Commons Zero "No rights reserved" data waiver (CC0 1.0 Public domain dedication).
