# Supplementary material for: Metformin improves FOXP3 mRNA expression through suppression of interferon gamma levels in pristane-induced murine models of lupus
Source: F1000Res. 2021 Jun 11;9:342. Originally published 2020 May 11. [Version 2] doi: 10.12688/f1000research.23471.2 (PMC8327221; doi:10.12688/f1000research.23471.2)
Supplement: Peer review history

# Peer review: Reviewer response for version 2

**DOI:** 10.5256/f1000research.56857.r90607

**Published:** 2021-08-06

**Authors:** Mirza Zaka Pratama

**Affiliations:** 1 Rheumatology and Immunology Division, Department of Internal Medicine, Faculty of Medicine, Universitas Brawijaya, Malang, Indonesia

This was an interesting study that showed the immunoregulatory effects of metformin in pristane-induced lupus mice.

 The study design and methods were also well written. However, I have a few minor suggestions regarding the study:

1. In the discussion section, I suggest not to put the results data anymore because they will overlap with the results section. Rather than using the data in the discussion, I suggest putting the resume or conclusion of the analysis, this way it will not overlap with the results section and make the discussion more concise.
2. Rather than using the correlation analysis for assessing the relationship and causality between the IFN gamma and FOXP3 mRNA changes, I suggest using the regression analysis that can show better results for explaining the causal-effect relationship
3. Although there are no significant differences for the histological scoring between the intervention groups, please provide the data in the results section so the reader can understand better

 Overall, this is a good study, and the authors already provided and presented the data well in this paper.

Is the work clearly and accurately presented and does it cite the current literature?

Yes

If applicable, is the statistical analysis and its interpretation appropriate?

Partly

Are all the source data underlying the results available to ensure full reproducibility?

Yes

Is the study design appropriate and is the work technically sound?

Yes

Are the conclusions drawn adequately supported by the results?

Yes

Are sufficient details of methods and analysis provided to allow replication by others?

Yes

Reviewer Expertise:

rheumatology, basic immunology, clinical immunology

I confirm that I have read this submission and believe that I have an appropriate level of expertise to confirm that it is of an acceptable scientific standard.

# Peer review: Reviewer response for version 2

**DOI:** 10.5256/f1000research.56857.r87397

**Published:** 2021-07-30

**Authors:** Shuang Ye

**Affiliations:** 1 Department of Rheumatology, Renji Hospital South Campus, Shanghai Jiaotong University School of Medicine, Shanghai, China

The revision looks good. The authors have clearly delivered the message that the route of administration of metformin is apparently important. I have no further comments.

Is the work clearly and accurately presented and does it cite the current literature?

Yes

If applicable, is the statistical analysis and its interpretation appropriate?

Partly

Are all the source data underlying the results available to ensure full reproducibility?

Yes

Is the study design appropriate and is the work technically sound?

Partly

Are the conclusions drawn adequately supported by the results?

Partly

Are sufficient details of methods and analysis provided to allow replication by others?

Yes

Reviewer Expertise:

NA

I confirm that I have read this submission and believe that I have an appropriate level of expertise to confirm that it is of an acceptable scientific standard.

# Response

**Published:** 2021-07-31

**Authors:** Stevent Sumantri

**Affiliations:** Universitas Pelita Harapan, Indonesia

Dear Dr Shuang Ye,

 Many thanks for your kind review and support. Looking forward to being able to work together with your team in Lupus research.

 Best regards,

 Stevent Sumantri, MD, PhD, DAA

# Peer review: Reviewer response for version 2

**DOI:** 10.5256/f1000research.56857.r87398

**Published:** 2021-07-27

**Authors:** Erin Taylor

**Affiliations:** 1 Department of Physiology and Biophysics, University of Mississippi Medical Center, Jackson, MS, USA

I have no further comments. The authors have revised the manuscript satisfactorily.

Is the work clearly and accurately presented and does it cite the current literature?

Partly

If applicable, is the statistical analysis and its interpretation appropriate?

No

Are all the source data underlying the results available to ensure full reproducibility?

Yes

Is the study design appropriate and is the work technically sound?

Yes

Are the conclusions drawn adequately supported by the results?

Yes

Are sufficient details of methods and analysis provided to allow replication by others?

Yes

Reviewer Expertise:

Lupus, autoimmunity, immunology, renal physiology, hypertension.

I confirm that I have read this submission and believe that I have an appropriate level of expertise to confirm that it is of an acceptable scientific standard.

# Response

**Published:** 2021-07-31

**Authors:** Stevent Sumantri

**Affiliations:** Universitas Pelita Harapan, Indonesia

Dear Dr Erin Taylor,

 Many thanks for the inputs and approval. Looking forward to future collaborations.

 Best regards,

 Stevent Sumantri, MD, PhD, DAA

# Peer review: Reviewer response for version 1

**DOI:** 10.5256/f1000research.25902.r83368

**Published:** 2021-04-27

**Authors:** Erin Taylor

**Affiliations:** 1 Department of Physiology and Biophysics, University of Mississippi Medical Center, Jackson, MS, USA

In the manuscript by Sumantri *et al*., the authors evaluated metformin in the pristane-inducible model of SLE. The authors tested both oral gavage and intraperitoneal administration of metformin as a treatment. Lupus was induced by a single ip injection of pristane into female BALB/c mice, after which lupus was allowed to develop for 120 days. At this point, mice were given vehicle, oral metformin, or IP metformin daily for 60 days. The experimental endpoints were as follows: the expression of mRNA for the Treg-associated transcription factor FoxP3 and circulating IFN-ɣ, both of which were assessed using blood collected from the tail vein. In addition, and histological examination of H&E stained kidneys was performed. The administration of pristane resulted in an increase in circulating IFN-ɣ and a decrease in the expression FoxP3, and treatment with metformin decreased IFN-ɣ and increased FoxP3 expression. The authors also conclude that treatment with pristane leads to renal injury, including glomerular changes, but minimal mesangial expansion. Treatment with metformin did not lead to significant changes in renal injury. Overall, this manuscript is written clearly, and the methods would be easy to follow if one were to repeat these experiments.

 One specific concern is the statistical analyses that are used. It is stated that t-tests were used; however, there are five experimental groups. I suggest that the statistical tests be redone using one-way ANOVA, followed by Tukey’s post test for multiple comparisons. Another issue/point of confusion that I have is with the presentation of the data in the Figures and Tables. In both Figure 1 and Figure 2, I suggest adding asterisks or other markers to indicate statistically significant differences among the groups. While it is stated in the text, it would be clearer if it was also on the graphs. Also, are the marks in Figure 1B, 1C, and 2A statistical outliers? Please indicate in the figure legends. In addition, Table 2 states that it is composed of IFN-ɣ and FoxP3 expression after intervention with metformin. While I think these are the data that are presented, the table says “post induction” as in Figure 1, which was referring to post treatment with pristane. I think that Table 2 should be relabeled with “post treatment” or something similar. Finally, the kidney specimens were scored, but those scores were not presented in any format. I would suggest presenting the scoring data in a graphical format in the manuscript. 

 The pristane model of SLE mimics human disease in many ways, including increases in IFN-production and dysregulation of T cell subsets. The data that the authors present suggest that metformin may improve the Th subset imbalances (increasing FoxP3, decreasing IFN-ɣ), but direct evidence of this is lacking. The findings could be strengthened by the addition of flow cytometry to assess Th subsets. In addition, the authors indicate that they did not measure immunoglobulins or autoantibodies in their animals. While data may be available in other studies, it would be a good additional piece of data for this study.

Is the work clearly and accurately presented and does it cite the current literature?

Partly

If applicable, is the statistical analysis and its interpretation appropriate?

No

Are all the source data underlying the results available to ensure full reproducibility?

Yes

Is the study design appropriate and is the work technically sound?

Yes

Are the conclusions drawn adequately supported by the results?

Yes

Are sufficient details of methods and analysis provided to allow replication by others?

Yes

Reviewer Expertise:

Lupus, autoimmunity, immunology, renal physiology, hypertension.

I confirm that I have read this submission and believe that I have an appropriate level of expertise to confirm that it is of an acceptable scientific standard, however I have significant reservations, as outlined above.

# Response

**Published:** 2021-04-27

**Authors:** Stevent Sumantri

**Affiliations:** Universitas Pelita Harapan, Indonesia

Dear Dr. Taylor,

 Many thanks for your inputs. I will revise the statistical methods and table as soon as possible. Regarding flow cytometry and antibody measurements, unfortunately, it was not possible at this time, but I will put it in discussion for further research plans. 

 Regards,

 Stevent Sumantri

# Peer review: Reviewer response for version 1

**DOI:** 10.5256/f1000research.25902.r63253

**Published:** 2020-05-26

**Authors:** Shuang Ye

**Affiliations:** 1 Department of Rheumatology, Renji Hospital South Campus, Shanghai Jiaotong University School of Medicine, Shanghai, China

The authors tried to illustrate the effect of metformin on pristane-induced mice in terms of IFN-γ levels and FOXP3 mRNA expression. However, the authors did not see the significant difference of renal histopathological score between intervention groups. It is better to provide evidence of effect of metformin on other manifestations, i.e. serological, in this model. The conclusion of "intravenous administration in human, could provide a novel route of administration...." could not be drawn from the data provided in the manuscript.

Is the work clearly and accurately presented and does it cite the current literature?

Yes

If applicable, is the statistical analysis and its interpretation appropriate?

Partly

Are all the source data underlying the results available to ensure full reproducibility?

Yes

Is the study design appropriate and is the work technically sound?

Partly

Are the conclusions drawn adequately supported by the results?

Partly

Are sufficient details of methods and analysis provided to allow replication by others?

Yes

Reviewer Expertise:

Our group carried out proof-of-concept trial and randomised placebo-controlled trial of metformin in lupus patients.

I confirm that I have read this submission and believe that I have an appropriate level of expertise to confirm that it is of an acceptable scientific standard, however I have significant reservations, as outlined above.

# Response

**Published:** 2020-05-26

**Authors:** Stevent Sumantri

**Affiliations:** Universitas Pelita Harapan, Indonesia

Dear Dr Shuang Ye,

Many thanks for your review on the research article. Could you kindly advise in which way we could further improve the quality of this article?

Regards, 

Stevent Sumantri MD

# Response

**Published:** 2020-06-07

**Authors:** shuang ye

**Affiliations:** Renji Hospital, China

1. The authors did not see the positive results of renal histopathology between intervention groups. We suggest to provide evidence of other manifestations, such as autoantibodies, in this model.

2. The conclusion of "intravenous administration in human, could provide a novel route of administration...." could not be drawn from the data provided in the manuscript, and should be deleted.

# Response

**Published:** 2021-04-27

**Authors:** Stevent Sumantri

**Affiliations:** Universitas Pelita Harapan, Indonesia

Dear Dr. Shuang Ye,

 Sorry for the late response; I will adjust the conclusion and writing of the reports according to our study limitations. Unfortunately, due to financial constraints, it was not possible to provide antibody data in our study; I will discuss the limitations in my revision. 

 Regards,

 Stevent Sumantri
